# Thoracic Endovascular Aortic Repair for Aortic Arch Aneurysms Using Physician-Modified Endovascular Graft with Pre-Crossing Cannulated Wires for the Left Common Carotid Artery Aortic Arch Aneurysms

**DOI:** 10.3400/avd.nmt.26-00007

**Published:** 2026-09-05

**Authors:** Shotaro Higa, Takaaki Nagano, Shogo Niizaki, Keita Miyaishi, Moriyasu Nakaema, Kojiro Furukawa

**Affiliations:** Department of Thoracic and Cardiovascular Surgery, University of the Ryukyus, Ginowann, Okinawa, Japan

**Keywords:** aortic arch, graft, thoracic endovascular aortic repair

## Abstract

Regarding thoracic endovascular aortic repair involving the aortic arch, fenestration is occasionally employed to maintain blood flow to aortic branches. Pre-cannulation can mitigate the risk of misalignment between the left subclavian artery fenestration and arch branches. However, the left common carotid artery is located in a tortuous area of the arch, making fenestration alignment difficult. We employed a novel pre-cannulation technique to prevent such misalignment in a 73-year-old patient. This technique represents a systematic approach to left common carotid artery fenestration alignment in thoracic endovascular aortic repair, with potential reproducibility in selected cases. This approach requires validation in larger cohorts.

## Introduction

Aortic branch preservation presents significant challenges in thoracic endovascular aortic repair (TEVAR) of the aortic arch, including stroke and upper limb ischemia. Consequently, various cerebral branch revascularization techniques have been developed, such as debranching, chimneying, fenestration/branching, and their combinations.^1)^ Proper alignment with the aortic branches is crucial during stent fenestration. A pre-cannulation technique has been described in which a catheter is pre-positioned through the fenestration in advance to facilitate accurate alignment of the left subclavian artery (LSA) with the fenestration of a physician-modified stent graft.^2,3)^ Chassin-Trubert et al. reported a double-fenestrated physician-modified stent-graft technique in which 1 fenestration was made for the brachiocephalic trunk and left common carotid artery (LCCA) together, and another fenestration was made for the LSA to preserve blood flow in the cervical branches. However, because the fenestrated area was larger than the cervical branches, there was a concern about the occurrence of Type Ia endoleak.^2)^ We report a technical refinement of the pre-cannulation approach designed to facilitate precise alignment of LCCA fenestrations.

## New Methods

**Fig. 1:**
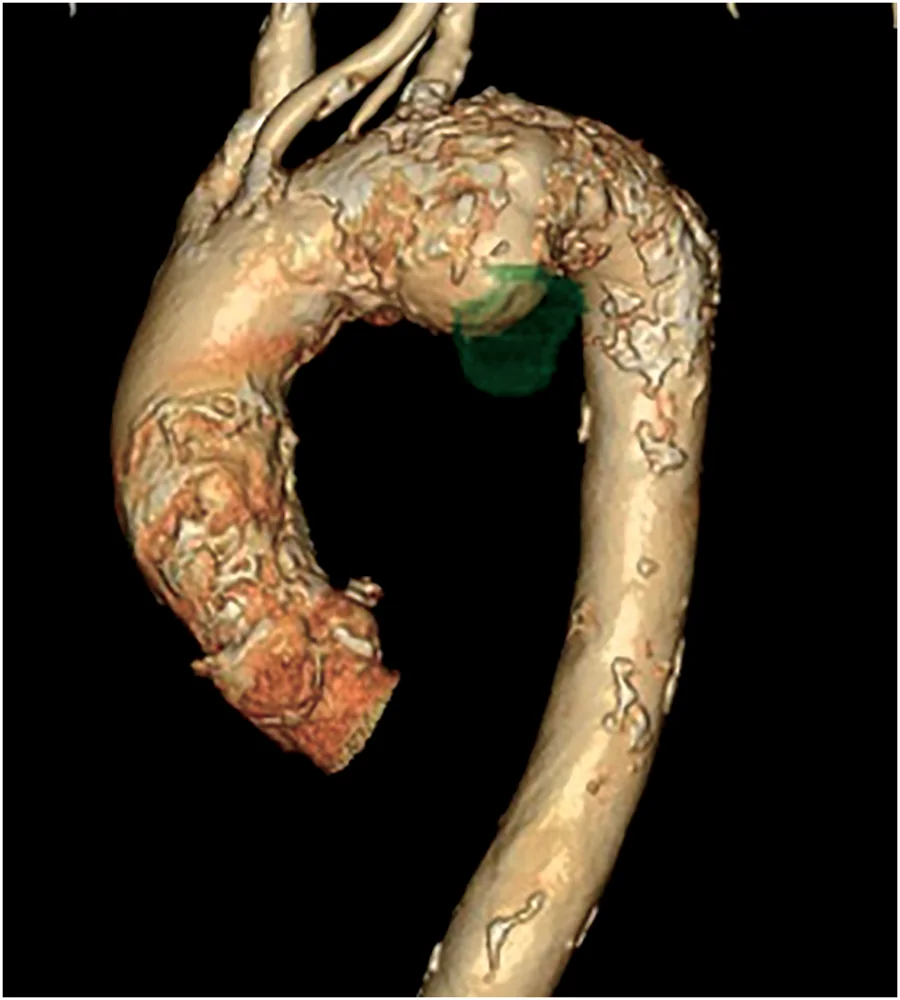
Contrast-enhanced CT shows a 60-mm saccular aneurysm. CT, computed tomography

A 73-year-old male patient undergoing maintenance dialysis was admitted after an aortic arch aneurysm was identified on computed tomography (CT). Contrast-enhanced CT demonstrated a 60-mm saccular aneurysm extending into the lesser curvature, along with marked calcification of the ascending aorta (**Fig. 1**). Although open surgical repair was recommended, the patient’s frailty and the inherently high perioperative risk associated with dialysis made the procedure prohibitively high risk. Regarding endovascular strategies, debranching TEVAR was deemed unsafe due to the severe calcification encompassing the ascending aorta (**Fig. 1**). Side-clamping or partial clamping in such a severely diseased ascending aorta poses an exceptionally high risk of catastrophic atheroembolism, stroke, or intraoperative retrograde Type A aortic dissection. Furthermore, the Najuta fenestrated stent graft was avoided because the patient had a steep aortic arch angle; in such anatomy, this device carries a risk of bird-beaking along the lesser curvature, which could lead to a Type Ia endoleak. Written informed consent was obtained for the procedure and publication of this case. The physician-modified endovascular stent graft (PMEG) and the off-label use of the Gore Viabahn VBX Balloon Expandable Endoprosthesis (W. L. Gore & Associates, Newark, DE, USA) for branch revascularization in fenestrated TEVAR were formally reviewed and approved by the Institutional Review Board of the University of the Ryukyus (approval nos.152 and 663).

**Fig. 2:**
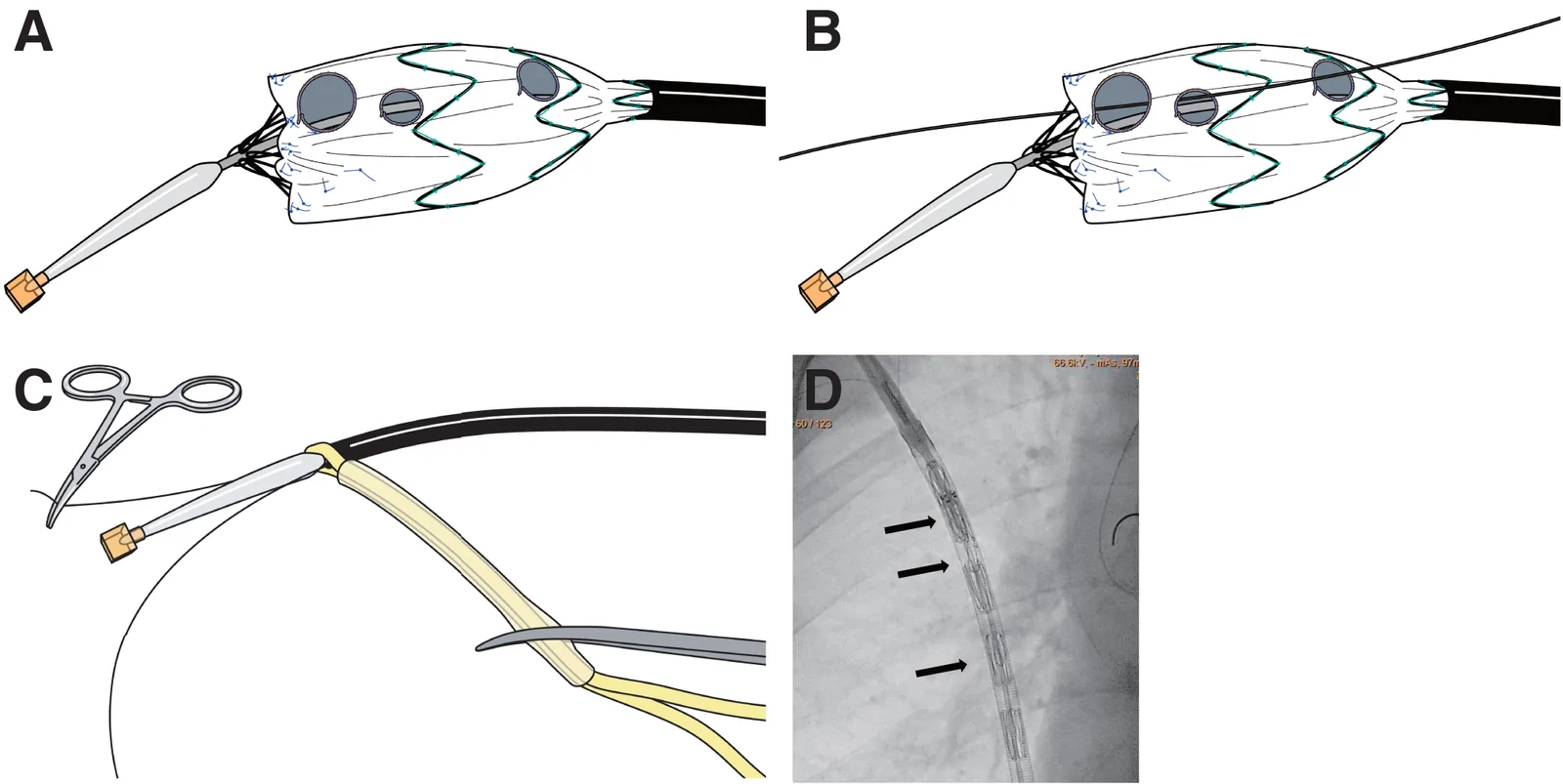
Physician-modified endovascular Zenith TX Alpha stent graft. (**A**) Three stent lows of a single Zenith TX Alpha stent graft are unsheathed, and 3 fenestrations are created. (**B**) A 0.025-inch Radifocus guidewire is advanced through the fenestration of the left common carotid artery. (**C**) The physician-modified endovascular graft is constrained by using ribbon loops that maintain the left common carotid artery-preloaded guidewire. (**D**) Fluoroscopic visualization of the fenestration (black arrows).

General anesthesia was administered, and vascular access was established using a cutdown approach at the right common femoral artery (CFA). Access was obtained via the LCCA through a cutdown of the left neck and through the left brachial artery via a cutdown at the left cubital fossa. A 6-Fr sheath was introduced into the right CFA, while 8-Fr sheaths were inserted into both the LCCA and left brachial artery. In addition, a 5Fr sheath was percutaneously inserted into the left CFA under ultrasound guidance, and heparin was administered. Three stent lows of a single Zenith TX Alpha stent graft (ZTA-P-42-38-225-W1) (Cook Medical, Bloomington, IN, USA) were partially unsheathed, and a 10 × 10-mm fenestration for the brachiocephalic artery was created 5 mm from the proximal position using a high-temperature fine-tip cautery (Geiger Medical Technologies, Council Bluffs, IA, USA). Subsequently, a 9 × 9-mm LCCA fenestration was established 5 mm distal to the previous fenestration, followed by creating a 10 × 10 mm fenestration of the LSA positioned 24 mm distal to the LCCA fenestration (**Fig. 2A**). One loop of an Atrieve snare catheter (Sugan, Osaka, Japan) was secured with a single interrupted 5-0 polypropylene suture to facilitate fluoroscopic visualization. Fenestration positioning was determined using 3-dimensional CT.

A 0.025-inch Radifocus guidewire (Terumo, Tokyo, Japan) was advanced through the LCCA fenestration, and the ribbon surrounding the partially deployed and released stent graft was tightened to facilitate retraction into the delivery catheter (**Fig. 2B** and **2C**). The stent graft was subsequently assessed for twisting (**Fig. 2D**). A Relay NBS stent graft (AR-N3615432) (Terumo) was inserted via the right CFA and positioned distal to the aneurysm for distal landing. Following stent-graft system removal, a 20-Fr DrySeal sheath (W. L. Gore & Associates) was inserted.

A 0.035-inch Radifocus guidewire was advanced from the 8-Fr LCCA sheath into the abdominal aorta, and pull-through was established using a snare catheter inserted from the 20-Fr DrySeal sheath. A 4-Fr JR catheter (Goodman Japan, Tokyo, Japan) was advanced from the LCCA and exteriorized along the Radifocus guidewire positioned from the right CFA; the 0.035-inch Radifocus guidewire was then removed. The other end of the 0.025-inch Radifocus guidewire that had been passed through the fenestration was inserted through a JR40 catheter exiting the right CFA and secured externally through the LCCA.

After removal of the DrySeal sheath, the JR catheter and the 0.025-inch Radifocus guidewire that had been housed within the fenestrated stent graft were retracted into the stent-graft sheath. The stent graft was then introduced via the right CFA and advanced over a pull-through guidewire, taking care to avoid entanglement with the TX Alpha stent graft. A 0.025-inch guidewire, introduced from the 8-Fr LCCA access into the aorta, externalized from the right CFA at procedure initiation, and kept taut by gentle traction to its ends, was used to accurately mark the distal border of the LCCA ostium.

**Fig. 3:**
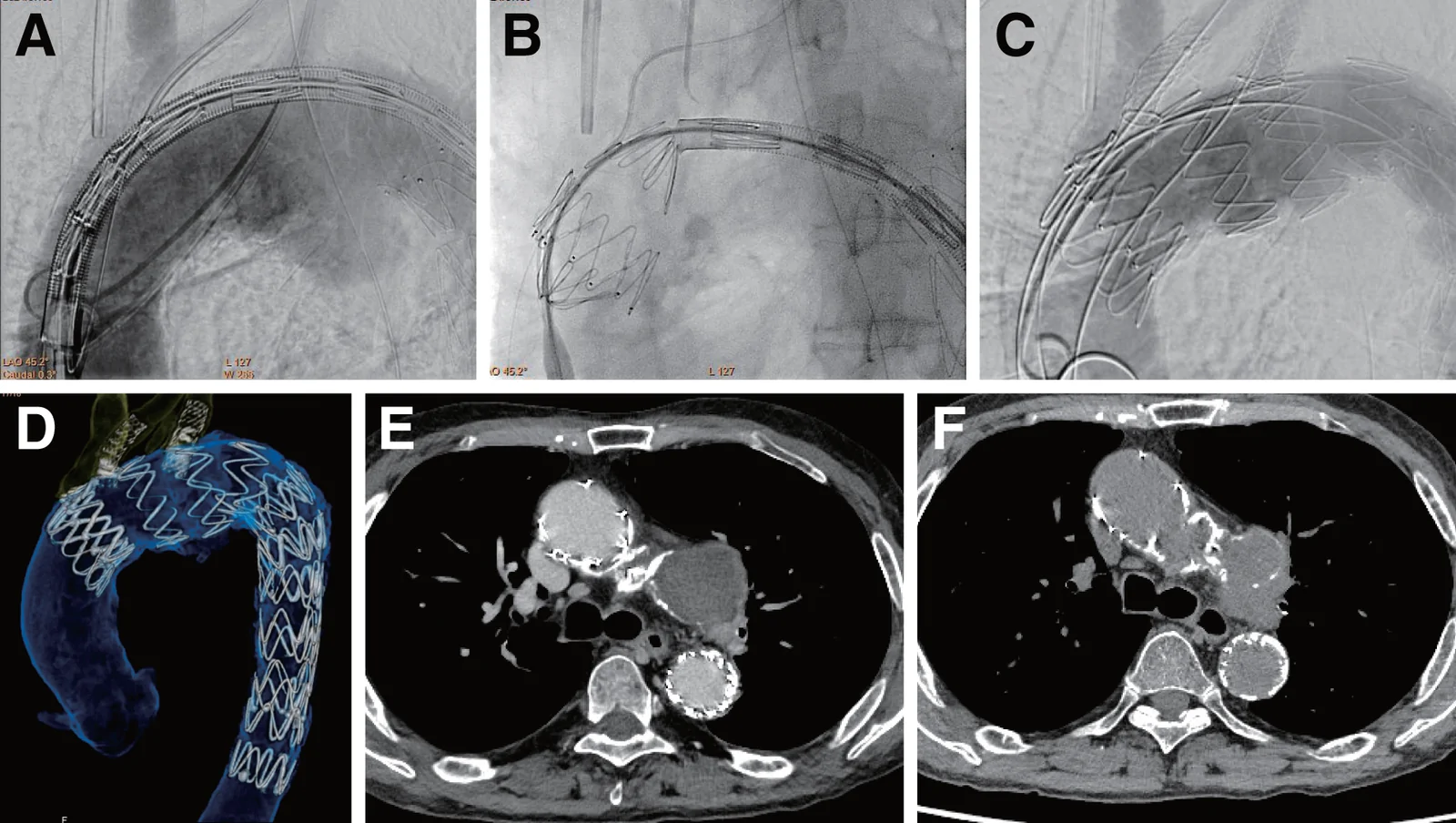
Intraoperative findings. (**A**) DSA before deployment of the physician-modified endovascular Zenith TX Alpha stent graft. (**B**) The device is deployed into the fenestration of the left common carotid artery, and a 4-Fr catheter is tried to cannulate into the fenestration. (**C**) Final DSA after VBX stent-graft deployment. (**D**) Postoperative contrast-enhanced CT shows no endoleaks. (**E**) Postoperative contrast-enhanced CT (axial). (**F**) CT at 18 months postoperatively demonstrating favorable sac regression. DSA, digital subtraction angiography; CT, computed tomography

Digital subtraction angiography (DSA) was performed to confirm the cervical branch positioning (**Fig. 3A**). Blood pressure was maintained at 80–90 mmHg using antihypertensive agents during deployment, and the device was deployed into the LCCA fenestration. An attempt was made to advance a 4-Fr JR catheter through the fenestration (**Fig. 3B**); however, it encountered interference with the center bar, leading to deployment into the LSA fenestration. The 4-Fr JR catheter was then advanced from the 8-Fr sheath of the brachial artery and successfully passed through the LSA fenestration. A Rosen guidewire (Cook Medical) was introduced, followed by placement of an Armada balloon (12 mm) (Abbott Laboratories, Abbott Park, IL, USA) within the fenestration; the balloon was then inflated securely, after which the stent-graft was fully deployed.

Subsequently, a 4-Fr catheter was reintroduced through the LCCA fenestration, and another Rosen guidewire was positioned. An Armada balloon (10 mm) was used for balloon inflation. A VBX stent graft (10 and 39 mm) was advanced from the LCCA, whereas a second VBX stent graft (11 and 39 mm) was advanced from the left brachial artery and successfully deployed. The final DSA showed only a minor type Ⅳ endoleak (**Fig. 3C**), which was shown to have resolved on postoperative contrast-enhanced CT (**Fig. 3D**). At the 18-month follow-up, the aneurysm showed a tendency to shrink (**Fig. 3E** and **3F**).

## Discussion

Fujimura et al. reported that only 5%–41% of patients with arch aneurysms are anatomically suitable for TEVAR using the fenestrated/branched endovascular grafts.^4)^

Although devices such as the Zenith Fenestrated stent graft (Cook Medical) for complex thoracoabdominal aortic aneurysms are available in the United States, many surgeons use alternative strategies such as PMEGs. Endovascular treatment in patients with complex anatomy would benefit from the broader publication of PMEG.^5)^ Once the technique is established, PMEGs are used more frequently worldwide due to restrictions on regional availability and time limits for urgent procedures.

During the PMEG procedure, the positioning of the stent-graft fenestration site is determined through a detailed analysis of 3-dimensional images generated using contrast-enhanced CT. A significant challenge lies in aligning the fenestration site with the cervical branch, which is facilitated by using radiopaque markers. Lounes et al. reported a pre-cannulation technique that more accurately directs the fenestration site toward the LSA.^6)^ However, because the brachiocephalic artery and LCCA are combined into a single large fenestration, potential leakage may be a concern. If 3 separate fenestrations are created, the pre-cannulation technique at the LSA allows its proper alignment and corresponding fenestration. However, if the LCCA and its fenestration are misaligned, adjusting their position is challenging.

Our technique extends the pre-cannulation concept to the LCCA, which presents unique anatomical challenges. Specifically, the LCCA often arises from a tortuous segment of the aortic arch, making precise alignment more demanding than with the LSA. To address this, we established a reliable reference point for device deployment by pre-cannulating the LCCA fenestration before loading the stent graft. The selection of the LCCA as the access site for this pre-cannulated wire technique represents a strategic refinement to enhance the reproducibility of this technique, supported by several considerations. First, the common carotid artery is located superficially in the neck, allowing for easy access via a limited cutdown with minimal tissue dissection. Furthermore, compared to the subclavian artery, the LCCA typically possesses a more robust vessel wall and less tortuosity, which significantly reduces the risk of vascular injury during sheath insertion—a critical advantage for patients on dialysis with fragile or calcified vessels. Additionally, the LCCA provides a relatively direct trajectory to the aortic arch, facilitating precise guidewire and catheter manipulation. This straight path is particularly advantageous for maintaining stable cannulation within the fenestration during stent-graft deployment. Finally, the transcarotid approach has been well-validated in transcatheter aortic valve implantation (TAVI) as a reliable alternative when femoral or subclavian access is unsuitable. Overtchouk et al. reported favorable outcomes with transcarotid TAVI, demonstrating low rates of stroke and access-site complications.^7)^ This established safety profile further justifies the use of carotid access in complex endovascular procedures such as the one described here.

Our pre-cannulation technique on the LCCA prevents misalignment between the artery and fenestration, reducing guidewire kinking by facilitating the insertion of a 0.025-inch Radifocus guidewire into the 4-Fr JR catheter, which is withdrawn from the body via the femoral artery.

In this instance, the bar of the guidewire lumen in the delivery system obstructs the fenestration, complicating the process of guiding the guidewire into the opening. Further unsheathing may have permitted fenestration expansion, thereby facilitating guidewire guidance. Even in the event of failed cannulation into the LCCA, stent-graft migration can be averted. The device can be stabilized by first engaging the LSA fenestration and subsequently completing the deployment while securing the fenestration with a balloon. Although the Najuta fenestrated stent graft (Sumitomo Bakelite, Tokyo, Japan) is a commercially available option, its large fenestrations remain a concern; Sato et al. reported a Type Ia endoleak rate of 27.8% associated with its use.^8)^ In contrast, our technique allows for smaller, more precisely tailored fenestrations, which is expected to reduce the incidence of endoleaks. Furthermore, compared to the Najuta device, the Zenith TX Alpha stent-graft features a smaller sheath profile, potentially expanding the eligibility for patients with narrow or challenging access vessels. In conclusion, we present a technical refinement of the pre-cannulation technique for LCCA fenestration in triple-fenestrated PMEG for aortic arch aneurysms. This approach provides a systematic method for achieving accurate alignment of the LCCA fenestration in tortuous arch anatomy. Although this single case demonstrates technical feasibility and favorable early outcomes, further experience in additional cases is necessary to establish the reproducibility, safety profile, and long-term durability of this technique.

## Declarations

### Data availability

Data sharing is not applicable to this article as no datasets were generated or analyzed during the current study.
